# Vitamin C Alleviates Heat-Stress-Induced Damages in Pig Thoracic Vertebral Chondrocytes via the Ubiquitin-Mediated Proteolysis Pathway

**DOI:** 10.3390/antiox13111341

**Published:** 2024-11-01

**Authors:** Xiaoyang Yang, Yabiao Luo, Mingming Xue, Shuheng Chan, Yubei Wang, Lixian Yang, Longmiao Zhang, Yuxuan Xie, Meiying Fang

**Affiliations:** 1 China Department of Animal Genetics and Breeding, National Engineering Laboratory for Animal Breeding, MOA Key Laboratory of Animal Genetics and Breeding, Beijing Key Laboratory for Animal Genetic Improvement, State Key Laboratory of Animal Biotech Breeding, Frontiers Science Center for Molecular Design Breeding, College of Animal Science and Technology, China Agricultural University, Beijing 100193, China; b20213040325@cau.edu.cn (X.Y.); yabiaoluo@cau.edu.cn (Y.L.); 20203040297@cau.edu.cn (M.X.); shubert@cau.edu.cn (S.C.); ylx123@cau.edu.cn (L.Y.); 2021304030324@cau.edu.cn (Y.X.); 2 China Sanya Institute, China Agricultural University, Sanya 572025, China; yvpek@cau.edu.cn (Y.W.); b20233040431@cau.edu.cn (L.Z.)

**Keywords:** vitamin C, weighted gene co-expression network analysis, ubiquitin-mediated proteolysis, ubiquitin protein ligase E3A, cell damage

## Abstract

Heat stress can impair organismal growth by inducing ubiquitination, proteasome-mediated degradation, and subsequent cellular damage. Vitamin C (VC) has been shown to potentially mitigate the detrimental effects of abiotic stresses on cells. Nevertheless, the impact of heat stress on growth plate chondrocytes remains unclear, and the underlying protective mechanisms of VC in these cells warrant further investigation. In this study, we focused on pig thoracic vertebral chondrocytes (PTVCs) that are crucial for promoting the body’s longitudinal elongation and treated them with 41 °C heat stress for 24 h, under varying concentrations of VC. Our findings reveal that, while oxidative stress induced by heat triggers apoptosis and inhibits the ubiquitin-mediated proteolysis pathway, the addition of VC alleviates heat-stress-induced oxidative stress and apoptosis, mitigates cell cycle arrest, and promotes cellular viability. Furthermore, we demonstrate that VC enhances the ubiquitin-proteasome proteolysis pathway by promoting the expression of ubiquitin protein ligase E3A, which thereby stabilizes the ubiquitin-mediated degradation machinery, alleviates the apoptosis, and enhances cell proliferation. Our results suggest the involvement of the ubiquitin-mediated proteolysis pathway in the effects of VC on PTVCs under heat stress, and offer a potential strategy to make use of VC to ensure the skeletal growth of animals under high temperature pressures in summer or in tropical regions.

## 1. Introduction

Heat stress is a significant environmental factor that adversely impacts optimal pig production efficiency [1]. Elevated temperatures exert detrimental effects on animal growth, especially within intensive livestock farming systems [2,3]. Body length, a commonly utilized metric for evaluating the body type of pigs and other livestock, is closely associated with meat yield and serves as a critical parameter for optimizing livestock production performance. The elongation of an animal’s body length can be achieved by extending individual vertebrae, which develop through the process of endochondral ossification of the growth plate cartilage [4]. Therefore, the proliferation and differentiation of growth plate chondrocytes are essential for facilitating longitudinal vertebral elongation. External temperature fluctuations can directly influence bone growth [5]. However, the effect of high-temperature heat stress on the growth plate chondrocytes in pigs has yet to be reported.

Vitamin C (VC) is a natural antioxidant that prevents lipid peroxidation by reducing the production of free radicals in lipids and thereby aiding in their elimination [6,7]. In humans, VC is widely recognized for its antioxidant properties, including its roles in anti-aging and protection against oxidative stress toxicity, and it is commonly used by athletes to neutralize exercise-induced free radicals [8]. In livestock, VC is extensively utilized to mitigate productivity losses caused by heat stress and to enhance meat quality [9]. However, the role of VC in protecting the chondrocytes of the thoracic vertebral growth plate of pigs from heat stress injury needs to be further explored.

Ubiquitin-mediated proteolysis is a fundamental protein degradation pathway in cells, playing a critical role in various cellular biological processes, including cell cycle regulation, signal transduction, DNA repair, immune response, and quality control [10,11]. This mechanism primarily operates by conjugating small ubiquitin proteins to target proteins, forming ubiquitin–protein complexes that are subsequently recognized and degraded by proteolytic enzymes [10,11]. Ubiquitin-mediated proteolysis has been extensively studied for its role in inhibiting cancer cell cycles, among other functions [12]. Heat stress can induce protein misfolding within cells, and the ubiquitin-mediated proteolysis pathway is crucial for degrading proteins that fail to fold correctly within the endoplasmic reticulum [13].

This study aims to investigate the physiological changes in chondrocytes within the growth plates of pig thoracic vertebrae under heat stress conditions and to elucidate the potential pathways in response to VC addition. Understanding the role of VC in heat stress alleviation is essential for preventing and addressing growth retardation caused by elevated temperatures.

## 2. Materials and Methods

### 2.1. Isolation and Culture of Primary Pig Thoracic Vertebral Chondrocytes

Using enzyme digestion, pig thoracic vertebral chondrocytes (PTVCs) were isolated from the thoracic vertebral cartilage tissues of three neonatal Yorkshire pigs in the Beijing pig breeding farm (Beijing, China). Cartilage samples were dissected into millimeter-sized fragments and incubated in DMEM/F12 medium supplemented with 2% fetal bovine serum (FBS), 3% penicillin-streptomycin, 1% amphotericin B, and 0.2% collagenase II at 37 °C for 8 h. Following incubation, the mixture was filtered through a 40-μm nylon cell strainer and centrifuged at 300× *g* for 10 min. The collected cells were then cultured in DMEM/F12 containing 10% FBS and 1% penicillin–streptomycin at 37 °C in 5% CO_2_ atmosphere. VC (Sigma-Aldrich, St. Louis, MO, USA) was added to the culture at a final concentration of 50 μM, 100 μM, 200 μM, and 400 μM, respectively, and the cells were cultured for 24 h. ML323 (Beyotime Bio, Shanghai, China) was added to the culture at a final concentration of 30 μM.

### 2.2. Detection of Intracellular Reactive Oxygen Species

The Reactive Oxygen Species (ROS) Assay Kit (Beyotime Bio, Shanghai, China) was utilized for the detection of ROS. Cells were seeded onto 12-well plates and incubated with VC at 41 °C (heat treatment) and 37 °C (control) for 24 h (n = 4). After the cell culture medium was removed, DCFH-DA, diluted 1:1000 in serum-free culture medium, was added to the cells. Following an incubation at 37 °C for 20 min, the cells were washed three times with serum-free culture medium and subsequently observed directly under a laser scanning confocal microscope (Echo Laboratories, San Diego, CA, USA) for fluorescence.

### 2.3. Biochemical Analysis

After pretreatment with the specified drug and temperature, PTVCs were collected and lysed (n = 4). The experimental protocol for detecting intracellular malondialdehyde (MDA) was conducted as follows. Cells were lysed using Western and IP Cell Lysis Buffer (Beyotime Bio, Shanghai, China). The lysate was then centrifuged at 12,000× *g* for 10 min to obtain the supernatant, which was subsequently used for MDA measurements. The protein concentration in the supernatant was determined using the BCA Protein Assay Kit (Beyotime Bio, Shanghai, China). Following the instructions of the Lipid Peroxidation MDA Assay Kit (Beyotime Bio, Shanghai, China), standards, absolute ethanol, thio-barbituric acid reagent, and samples were added to the assay tubes. Additionally, total antioxidant capacity (TAC) was measured using the Total Antioxidant Capacity Assay Kit with the ABTS method (Beyotime Bio, Shanghai, China).

### 2.4. Cell Counting Kit-8 Assay

PTVCs were seeded into 96-well plates and cultured in a basal medium (n = 4). Cell proliferation was monitored at 41 °C and 37 °C for 24 h after adding VC using the Cell Counting Kit-8 (Beyotime Bio, Shanghai, China) following the manufacturer’s protocol. After 1 h of incubation, absorbance was measured at 450 nm using a SpectraMax^®^ i3x Multi-Mode Microplate Reader (Molecular Devices Corporation, Sunnyvale, CA, USA).

### 2.5. 5-Ethynyl-2′-deoxyuridine Assay

PTVCs were seeded into 12-well plates and cultured in a basal medium (n = 4). After 24 h heat stress (41 °C), the cells were stained with Alexa Fluor 555 for 2 h using the BeyoClick™ EdU Cell Proliferation Kit (Beyotime Bio, Shanghai, China) following the manufacturer’s protocol. Cell nuclei were stained in blue, and EdU-positive cells were stained in red. The cells were then observed and photographed using an Echo Revolve fluorescence microscope (Echo Laboratories, San Diego, CA, USA).

### 2.6. Cell Apoptosis

The Annexin V-FITC Apoptosis Detection Kit (Beyotime Bio, Shanghai, China) was used to label the membranes and nuclei of apoptotic cells. PTVCs were collected and resuspended after pretreatment (n = 4). The samples were mixed with Annexin V-FITC and propidium iodide (PI) and incubated in the dark for 5–10 min. Flow cytometry data was then acquired using a BD FACSCalibur flow cytometer (BD Biosciences, San Diego, CA, USA).

### 2.7. Cell Cycle Assay

PTVCs were seeded in 6-well plates and cultured in basal medium (n = 4). After 24 h at 41 °C, cells were harvested by centrifugation at 1000× *g* for 5 min. After washing with cold phosphate-buffered saline (PBS) (Gibco, Waltham, MA, USA), the cells were fixed in cold 70% ethanol at 4 °C overnight. The cells were then collected again by centrifugation at 1000× *g* for 5 min and incubated with propidium iodide staining solution (Beyotime Bio, Shanghai, China) at 37 °C for 30 min in the dark, following the manufacturer’s instructions. The cell cycle was analyzed using a BD FACSCalibur flow cytometer (BD Biosciences, San Diego, CA, USA).

### 2.8. Western Blot Analysis

PTVCs treated with 41 °C and VC were collected and lysed using radioimmunoprecipitation assay (RIPA) lysis buffer (Thermo Fisher Scientific, Waltham, MA, USA) (n = 4). Samples were boiled and denatured in loading buffer containing 5% 2-mercaptoethanol (Invitrogen, Waltham, MA, USA) and subjected to electrophoresis through a 10% SDS polyacrylamide gel. The separated proteins were transferred to a PVDF membrane (Bio-Rad, Los Angeles, CA, USA) and probed with primary antibodies for ubiquitin-protein ligase E3A (UBE3A) 1/500 (Beyotime Bio, Shanghai, China), β-Tubulin 1/2000 (Beyotime Bio, Shanghai, China) and the appropriate secondary antibody conjugated with horseradish peroxidase 1/2000 (Beyotime Bio, Shanghai, China). Bound proteins were detected using a BeyoECL Moon (Beyotime Bio, Shanghai, China). The results were visualized by scanning and then analyzed using Image J software (Version: 1.53).

### 2.9. Transcriptome Analysis

Total RNA was extracted using TRIzol reagent, followed by quantification and quality assessment with a Nanodrop spectrophotometer. Sixteen complementary DNA (cDNA) libraries were synthesized from four groups (n = 4) using SuperScript™ II Reverse Transcriptase (Invitrogen, Waltham, MA, USA) and subsequently subjected to paired-end sequencing on an Illumina HiSeq Novaseq™ 6000 platform. The sequenced reads were quality-controlled, filtered, and mapped to the pig reference genome (Sus scrofa 11.1, GenBank Assembly Accession: GCF_000003025.6) using HISAT2. Gene expression levels were calculated using featureCounts and normalized to transcripts per kilobase million (TPM). Differential gene expression analysis was conducted using the limma package in R, employing a linear model based on empirical Bayesian methods. The criteria for identifying differentially expressed genes (DEGs) were established at *p* < 0.05 and |log2 fold change| ≥ 1. Pathway enrichment analysis, including Kyoto Encyclopedia of Genes and Genomes (KEGG) and Gene Ontology (GO) annotations, was performed using the clusterProfiler R package (Jinan University, Guangzhou, China) [14]. Weighted Gene Co-expression Network Analysis (WGCNA) was utilized to identify hub mRNAs associated with the mitigation of heat stress by VC based on the expression levels of all identified mRNAs.

### 2.10. Quantitative Real-Time PCR

PTVCs were lysed using TRIzol reagent (TIANGEN, Beijing, China) for total RNA extraction (n = 4). To detect mRNA expression, 2 μg of total RNA from each sample was reverse-transcribed into complementary DNA (cDNA) using the FastKing RT Kit (with gDNase) (TIANGEN, Beijing, China) according to the manufacturer’s instructions. A SYBR Green-based quantitative real-time PCR (qRTP–CR) was performed in a 20 μL reaction volume containing 10 μL of 2× Universal SYBR Green Fast qPCR Mix (aBclonal, Wuhan, China), 8 μL of RNase-free water, 0.5 μL each of forward and reverse primers (10 μmol/L), and 1 μL of cDNA (approximately 300 ng). The qRT–PCR amplification protocol consisted of an initial denaturation at 95 °C for 15 min, followed by 40 cycles of denaturation at 94 °C for 20 s and annealing/extension at 60 °C for 34 s. Hypoxanthine phosphoribosyl-transferase 1 (*HPRT1*) was used as the internal reference gene. The primer sequences are presented in Table S1.

### 2.11. Statistical Analysis

The statistical analysis was conducted using SPSS version 22.0 (IBM, Armonk, NY, USA). To ascertain the significance of differences between the two groups, a non-paired t-test was employed. The examination of interaction effects was carried out through a 2 × 2 factorial design, with the application of analysis of variance using the univariate general linear model test. Polynomial contrast analysis was utilized to assess linear and quadratic response trends. The least significant difference test was implemented for multiple comparisons (* *p* < 0.05, significant; ** *p* < 0.01, highly significant). GraphPad Prism 9.0 was used for plotting the data, and all results were presented as mean ± SEM.

## 3. Results

### 3.1. Heat Stress Causes Oxidative Stress and Apoptosis of Pig Thoracic Vertebral Chondrocytes

**Figure 1:**
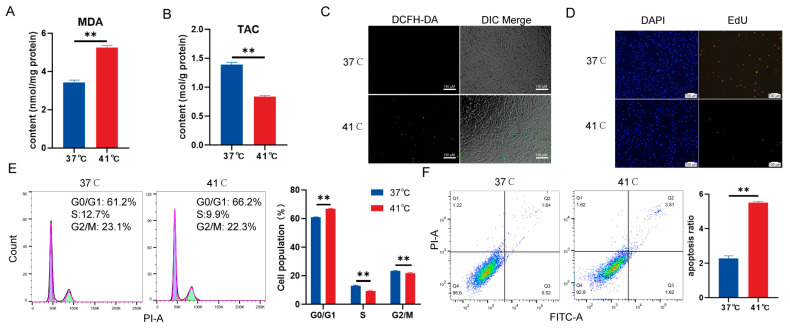
The damage to PTVCs by heat stress for induction of oxidative stress and inhibition of PTVC proliferation. Heat stress was applied by exposing the cells to 41 °C for 24 h. PTVCs cultured under 37 °C served as the controls. The intracellular MDA levels (**A**) and TAC (**B**) were evaluated between the control and heat stress. (**C**) ROS were detected using the DCFH-DA fluorescent dye. DIC: differential interference contrast (**D**) PTVC proliferation was determined by EdU staining after 24 h of heat stress. (**E**) Cell cycle analysis was conducted using flow cytometry following 24 h of heat stress. G0/G1: DNA pre-synthetic phase, S: stage of DNA synthesis, G2/M: DNA post-synthetic/mitosis phase, PI-A: propidium iodide (**F**). The effect of heat stress on apoptosis was detected by flow cytometry analysis, with Q1 indicating mechanical trauma, Q2 indicating late apoptosis, Q3 indicating living cells, and Q4 indicating early apoptosis. FITC: fluorescein 5-isothiocyanate. The bar chart shows the apoptosis rate, calculated as the sum of early and late apoptosis. All data were represented as mean ± SD (n = 4). Statistical significance between groups is indicated by “**” (*p* < 0.01). The experiment was repeated three independent times.

Culturing pig cells at 41 °C for 24 h effectively induces a heat stress response [15,16,17]. To investigate the response to oxidative stress, PTVCs were cultured at the control temperature (37 °C) and heat stress (41 °C) for 24 h. The levels of MDA were found to increase after exposure to heat stress (Figure 1A). In contrast, TAC levels were downregulated under heat stress (Figure 1B). Intracellular ROS levels in PTVCs were measured using DCFH-DA fluorescent dye, revealing a significant increase in fluorescence intensity under heat stress (Figure 1C and Figure S1A). EdU staining analysis demonstrated a marked reduction in the number of EdU-positive PTVCs in the 41 °C group compared to the 37 °C control group (Figure 1D and Figure S1B). Furthermore, flow cytometry analysis showed that 41 °C impeded the normal progression of the cell cycle, as evidenced by a significant increase in the G0/G1 phase and a substantial reduction in the S and G2/M phases (Figure 1E). Additionally, 41 °C was observed to increase the apoptosis rate of PTVCs (Figure 1F). These findings indicate that heat stress induces oxidative stress, reduces antioxidant capacity, inhibits cell cycle progression, promotes cell apoptosis, and ultimately suppresses cellular proliferation.

### 3.2. Vitamin C Relieves Heat-Stress-Induced Oxidative Stress and Apoptosis in Pig Thoracic Vertebral Chondrocytes

**Figure 2:**
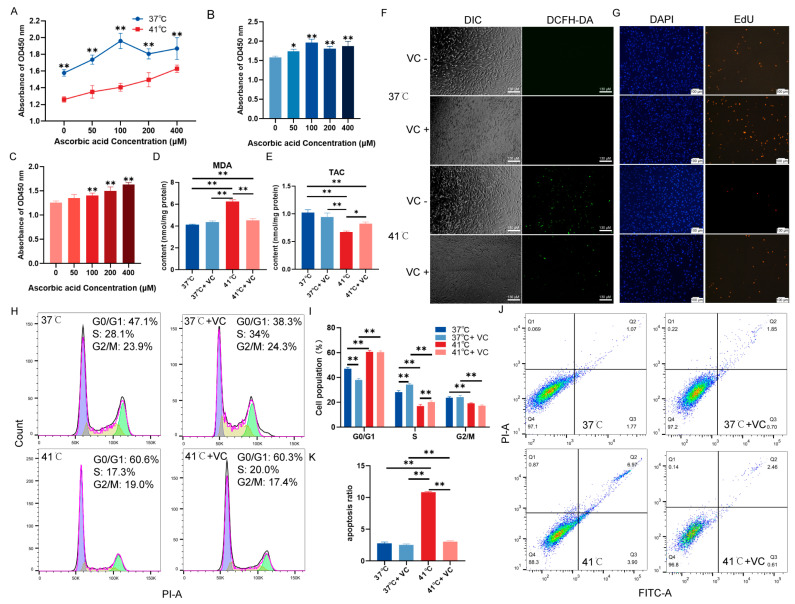
Alleviated heat-stress-induced oxidative stress and apoptosis in PTVCs after VC addition. (**A**) The viability of PTVCs treated with 50 μM, 100 μM, 200 μM, and 400 μM VC (i.e., ascorbic acid) is compared between 37 °C and 41 °C, based on CCK-8 assay. (**B**) The 50 μM, 100 μM, 200 μM, and 400 μM VC were added and compared for the performance of PTVC viability under 37 °C, based on CCK-8 assay. (**C**) The 50 μM, 100 μM, 200 μM, and 400 μM VC were added and compared for the performance of PTVC viability under 41 °C, based on CCK-8 assay. The intracellular MDA content (**D**) and TAC (**E**) were assessed under 100 μM VC and heat stress. (**F**) ROS levels were detected using DCFH-DA fluorescent dye after 24 h of heat stress and 100 μM VC treatment. DIC: differential interference contrast. (**G**) PTVC proliferation was determined by EdU staining after 24 h of heat stress and 100 μM VC treatment. (**H**) Cell cycle analysis of PTVCs was conducted after 24 h of heat stress using flow cytometry, and compared among the difference treatments in the three stages of G0/G1, S, and G2/M (**I**). G0/G1: DNA pre-synthetic phase, S: stage of DNA synthesis, G2/M: DNA post-synthetic/mitosis phase, PI-A: propidium iodide. (**J**,**K**) The effect of heat stress on apoptosis was detected by flow cytometry analysis, with Q1 indicating mechanical trauma, Q2 indicating late apoptosis, Q3 indicating living cells, and Q4 indicating early apoptosis. FITC: fluorescein 5-isothiocyanate. The bar chart shows the apoptosis rate, calculated as the sum of early and late apoptosis. All data was represented as mean ± SD (n = 4). Statistical significance between groups is indicated by “*” (*p* < 0.05) or “**” (*p* < 0.01). The experiment was repeated three independent times.

Heat stress reduced the proliferative capacity of PTVCs compared to the control at 37 °C, across all the VC concentrations (50 μM, 100 μM, 200 μM, and 400 μM) (Figure 2A). The addition of varying concentrations of VC enhanced the proliferative ability of PTVCs at 37 °C (Figure 2B). Notably, the most significant enhancement in cellular viability was observed at an VC concentration of 100 μM, so the VC concentration of 100 μM was selected for subsequent experiments. Under heat stress conditions, all concentrations of VC improved the proliferative capacity of PTVCs in a dose-dependent manner (Figure 2C). After adding 100 μM VC prior to heat stress, the group for 41 °C + VC exhibited significant reductions in MDA levels compared to the heat-stress-only group (41 °C) (Figure 2D). Meanwhile, there are significant increases in TAC activities (Figure 2E). Additionally, a significant reduction in ROS levels was observed (Figure 2F and Figure S1C). At 37 °C, supplementation with VC promotes PTVC proliferation, and mitigates the inhibitory effects of heat stress on their proliferation (Figure 2G and Figure S1D). Furthermore, compared to the heat-stress-only group, the heat-stress+VC group demonstrated a significant acceleration in cell cycle progression (Figure 2H,I). Additionally, this heat-stress+VC group exhibited a notable reduction in apoptosis levels (Figure 2J,K). These observations collectively suggest that VC alleviates oxidative damage induced by high temperatures in PTVCs, thereby promoting cellular proliferation.

### 3.3. Regulatory Pathways in Transcriptome Analysis

**Figure 3:**
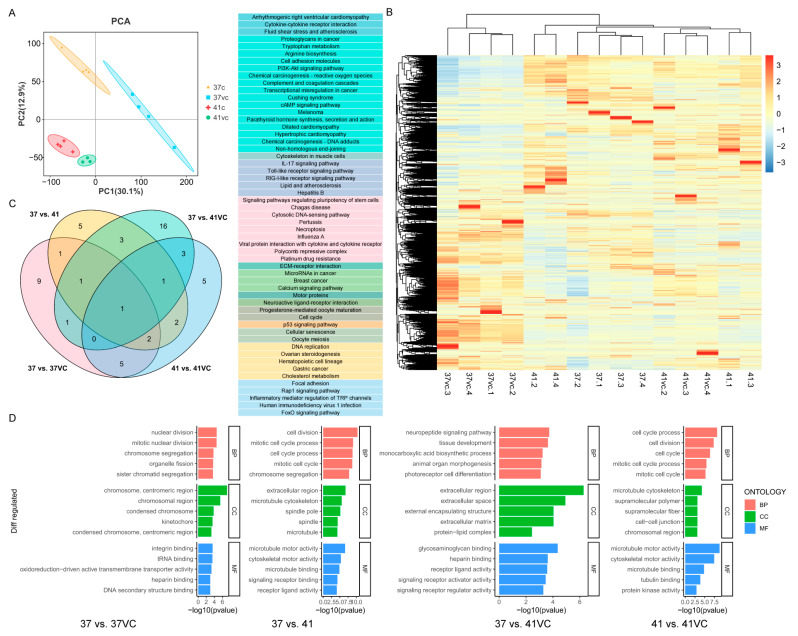
The gene patterns by using transcriptome analyses for treatments of VC and heat stress. (**A**) PCA of transcriptomics. (**B**) Unsupervised hierarchical clustering heatmaps of genes were detected in each treatment group. (**C**) Venn diagram showed the shared or unique KEGG pathways in four pairwise comparison sets. (**D**) GO enrichment analysis in four pairwise comparison sets.

To further investigate the role of VC in mitigating heat stress, we conducted a transcriptome analysis. The principal component analysis (PCA) of the transcriptome data revealed distinct separation among the four groups: 37 °C (37), 37 °C with VC (37VC), 41 °C (41), and 41 °C with VC (41VC) (Figure 3A). Hierarchical clustering analysis further demonstrated distinct gene expression patterns across the fifteen groups (Figure 3B). Subsequently, we performed a KEGG enrichment analysis to elucidate the underlying biological pathways. The pathway for motor proteins was significantly enriched in all the paired comparisons of 37 vs. 37VC, 37 vs. 41VC, 41 vs. 41VC, and 37 vs. 41 (Figure 3C). Pathways (e.g., ECM-receptor interaction) were enriched in 37 vs. 41, 37 vs. 41VC, and 41 vs. 41VC (Figure 3C). The cell cycle pathway was enriched in 37 vs. 41, 37 vs. 37VC, and 41 vs. 41VC, while DNA replication was uniquely enriched in 37 vs. 41 (Figure 3C). The cytosolic DNA-sensing pathway was uniquely enriched in 37 vs. 37VC (Figure 3C). These common KEGG pathways identified are associated with cell damage and cyto-dynamics. Additionally, GO enrichment analysis revealed that 37 vs. 41 was enriched in cell cycle processes, microtubule motor activity, and cytoskeletal motor activity (Figure 3D). The comparison of 37 vs. 37VC showed enrichment in nuclear division and DNA secondary structure binding, while 37 vs. 41VC was enriched in the neuropeptide signaling pathway and receptor-ligand activity (Figure 3D). Lastly, 41 vs. 41VC was enriched in cell cycle processes, microtubule cytoskeleton, and microtubule motor activity (Figure 3D). These findings suggest the strong cellular response to heat stress in pig cells and indicate that VC alleviates heat-stress-induced damages through pathways related to cell cycle regulation, signal transduction, and DNA repair.

### 3.4. The Ubiquitin-Mediated Proteolysis Signaling Pathway in Weighted Gene Co-Expression Network Analysis

**Figure 4:**
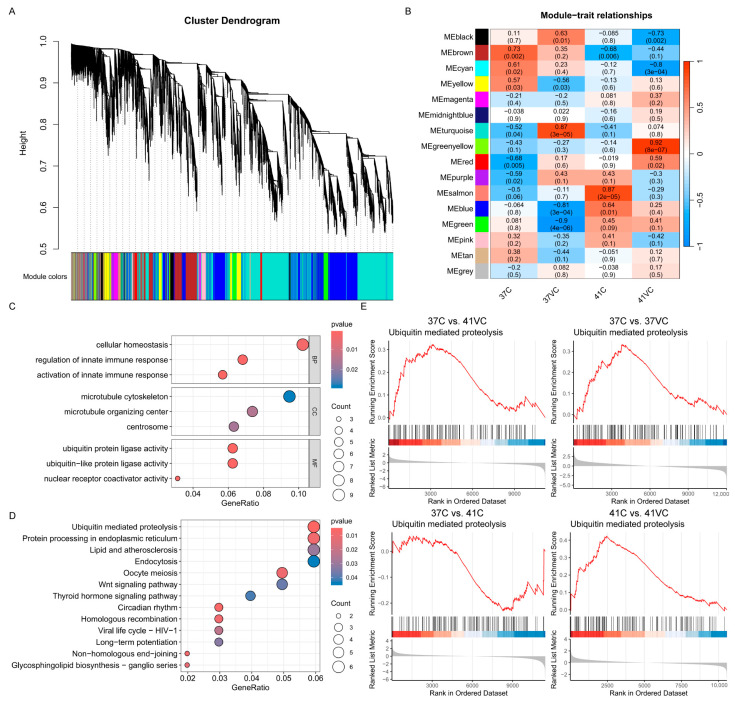
Gene modules by WGCNA. (**A**) The hierarchical clustering tree diagram displays 15 modules of co-expressed genes, with each module represented by a different color. (**B**) The module–trait relationship heatmap illustrates the associations between each module eigengene (rows) and the treatments (columns). (**C**) GO enrichment analysis of genes that are significantly positively associated with VC under heat stress. (**D**) KEGG enrichment analysis of genes that are significantly positively associated with VC under heat stress. (**E**) The genes in the ubiquitin-mediated proteolysis pathway are enriched in ranked gene list of the three comparison sets (37C vs. 41VC, 37C vs. 37VC, and 41C vs. 41VC) by gene set enrichment analysis (GSEA), but not in the comparison of 37C vs. 41C.

To gain a better understanding of the regulatory pathways through which VC alleviates cellular damage caused by heat stress, we employed WGCNA to identify relevant hub genes. We selected a soft-thresholding power value of 6 for network topology analysis, which was the minimal threshold required for the scale-free topology fit index curve to plateau, achieving an R^2^ cutoff of 0.85. This ensured the reliability and validity of our network construction (Figure S2A). Cluster analysis using topological overlap matrix (TOM) values resulted in the construction of 15 gene modules, which were partitioned using hybrid dynamic shear (Figure 4A). Four of these modules exhibited significant module-trait associations (*p* < 0.01), including the green-yellow (*p* = 8 × 10^−7^) and red (*p* = 0.02) modules, which were significantly positively associated with VC under heat stress, and the black (*p* = 0.002) and cyan (*p* = 3 × 10^−4^) modules, which were significantly negatively correlated with VC under heat stress (Figure 4B).

Using gene significance (GS) and module membership (MM) measurements, we identified hub genes with high correlations to VC under heat stress (|GS| > 0.8) and high correlations with the module eigengene and gene expression profiles (|MM| > 0.8) within the green-yellow and red modules, which are significantly positively associated with VC under heat stress (Figure S2B,C). A total of 123 hub genes were identified within these two modules (Table S2). Furthermore, we conducted functional enrichment analysis on these 123 hub genes via GO and KEGG. In the biological process (BP) category, the 41VC hub genes were primarily involved in cellular homeostasis and other functions. In the cellular component (CC) category, they were involved in the microtubule cytoskeleton and related structures. In the molecular function (MF) category, they were associated with ubiquitin protein ligase activity and other related functions (Figure 4C). According to KEGG analysis, these hub genes were particularly enriched in the ubiquitin-mediated proteolysis pathway and other related pathways (Figure 4D).

GSEA was used to demonstrate the overall dynamic alterations in gene sets related to the ubiquitin-mediated proteolysis process. GSEA plots revealed that VC significantly promoted the expression of this gene set, while heat stress exerted a suppressive effect (Figure 4E). Collectively, these observations suggest that both heat treatment and VC administration profoundly reshape the protein modification landscape of PTVCs, with changes likely associated with the activation of the ubiquitin-mediated proteolysis pathway. In subsequent experiments, we explored the alterations in ubiquitin-mediated proteolysis.

### 3.5. Verification of Genes and Proteins in the Ubiquitin-Mediated Proteolysis Signaling Pathway

**Figure 5:**
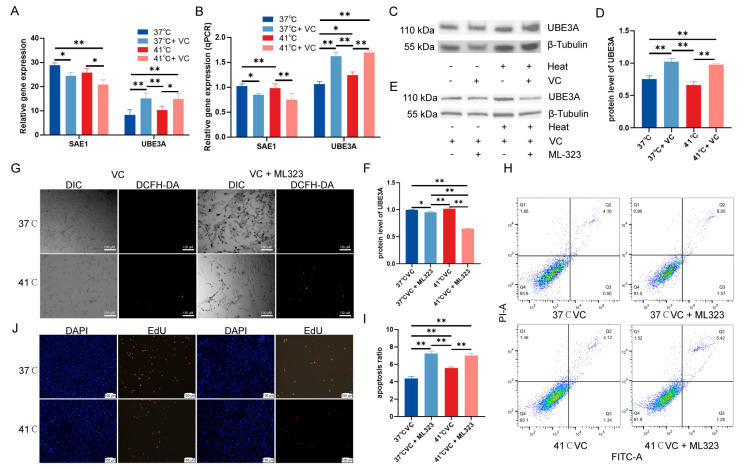
Validation of the ubiquitin-mediated proteolysis pathway in VC alleviation against heat stress. (**A**) The impact of heat stress and 100 μM VC on *SAE1* and *UBE3A* mRNA expression levels in PTVCs was assessed using RNA-seq. (**B**) qRT–PCR analysis was conducted to verify the effect of heat stress and 100 μM VC on *SAE1* and *UBE3A* mRNA expression levels in PTVCs. (**C**,**D**) UBE3A protein expression levels in PTVCs under heat stress or 100 μM VC. (**E**,**F**) UBE3A protein expression levels in PTVCs under heat stress, 100 μM VC and ML323. (**G**) Intracellular ROS under heat stress, 100 μM VC and ML323. DIC: differential interference contrast. DCFH-DA: fluorescent probe for ROS detection. (**H**) Proliferation of PTVCs determined by EdU after 24 h heat stress, VC and ML323. FITC: fluorescein 5-isothiocyanate. (**I**,**J**) The effect of heat stress on apoptosis was detected by flow cytometry analysis, with Q1 indicating mechanical trauma, Q2 indicating late apoptosis, Q3 indicating living cells, and Q4 indicating early apoptosis. PI-A: propidium iodide. The bar chart shows the apoptosis rate, calculated as the sum of early and late apoptosis. All data were represented as mean ± SD (n = 4). Statistical significance between groups is indicated by “*” or “**” (* *p* < 0.05; ** *p* < 0.01). The experiment was repeated three independent times.

To determine whether the protective effects of VC against heat stress are mediated through the ubiquitin-mediated proteolysis pathway, we conducted qTRP–CR and western blot to examine the expression of key genes involved in this pathway. Among the list of enriched genes in this WGCNA pathway (Table S3), we selected *SAE1* and *UBE3A* for validation. Our results indicate that the mRNA levels of *SAE1* were downregulated in the samples of heat-stress+VC (Figure 5A), while the mRNA levels of *UBE3A* were upregulated (Figure 5A). This trend was consistent with the expression patterns observed in the qRT–PCR analysis (Figure 5B). Furthermore, we validated these findings at the protein level, confirming that UBE3A protein expression was significantly upregulated in the samples of heat-stress+VC (Figure 5C,D). To further investigate the role of ubiquitin-mediated proteolysis, PTVCs were treated with the ubiquitin-protein inhibitor ML323. This treatment led to a reduction in ubiquitin-mediated proteolysis under heat stress, as evidenced by the decreased protein expression levels of UBE3A (Figure 5E,F). Notably, inhibition of this pathway did not result in significant changes in ROS levels (Figure 5G and Figure S1E), but it was associated with a marked increase in cell apoptosis (Figure 5H,I). When VC was supplemented in conjunction with ML323, its protective effect against heat stress was compromised, which in turn hindered the promotion of cell proliferation by VC under heat stress conditions (Figure 5J and Figure S1F). These findings suggest that VC exerts its protective effects against heat stress-induced cellular damage primarily through the ubiquitin-mediated proteolysis signaling pathway.

## 4. Discussion

External temperature can directly influence bone elongation by regulating chondrocytes in the growth plate [5]. It has been reported that heat stress can influence the extracellular matrix of pig chondrocytes [17]. Similarly, various cell types exposed to heat stress generate ROS, which subsequently induce cellular damage. For example, heat-induced oxidative stress in pig Sertoli cells triggers apoptosis [18], while heat stress inhibits the proliferation of pig skeletal muscle satellite cells and promotes their apoptosis [15]. Additionally, high temperatures have been found to impair chondrocyte differentiation in the tibial growth plate of chickens [19]. Our study is the first to demonstrate, in vitro, that heat stress leads to an upregulation of oxidative stress levels in chondrocytes from pig thoracic vertebral growth plates. This surge in ROS affects the cell cycle, promotes apoptosis, and ultimately impedes cell proliferation. Variations in ambient temperature within farming environments directly influence pig growth [20]. In the context of global warming, understanding the effects of high-temperature heat stress on the proliferation of chondrocytes in pig growth plates is crucial for interpreting its impact on pig body length traits.

VC plays a pivotal role in chondrocyte culture processes and is crucial in mitigating heat stress [21]. VC and sodium bicarbonate can enhance the antioxidant capacity of H9C2 cells by inducing the expression of heat shock proteins, thereby alleviating heat stress [22]. However, the role of VC in modulating chondrocyte proliferation and differentiation under varying temperatures remains unexplored in the literature. Stress-induced ROS production is critical in several key cellular events, including cell growth, apoptosis, and post-translational modification pathways [23]. Although cells possess various defense mechanisms, including enzymatic and non-enzymatic antioxidant systems, to counteract the adverse effects of oxidative stress and maintain redox homeostasis, excessive ROS generation can damage vital biomolecules such as proteins, lipids, and DNA, triggering a cascade of events that compromise cellular functions [24,25]. Here, we propose for the first time that the addition of VC during heat treatment of chondrocytes can significantly reduce oxidative stress induced by heat stress, thereby enhancing cell viability and proliferation capacity. This approach offers a novel strategy to mitigate the negative consequences of heat stress on chondrocyte biology.

In general, an independent biological process relies on intricate interactions involving functional gene networks. We employed WGCNA to identify key modules and hub genes related to VC under heat stress. This analytical approach seeks to identify co-expressed gene modules, explore the associations between gene networks and phenotypes of interest, and pinpoint the core genes within the network [26]. In our study, two modules were found to be significantly positively correlated with VC supplementation during heat stress, within which 213 genes were identified as hub genes. Enrichment analysis revealed that the pathways associated with these genes may represent critical pathways through which VC mediates the mitigation of cellular damage under heat stress. Among the pivotal signaling pathways, the Protein Processing in Endoplasmic Reticulum (PERK) signaling pathway has been extensively reported to underlie the mechanism of cellular damage caused by endoplasmic reticulum stress induced by heat stress [27,28,29]. Similarly, the enriched endocytic pathway also plays a significant role in the heat stress response. In yeast, ubiquitin-mediated endocytosis, which removes heat stress-induced damaged proteins from the plasma membrane, serves as a crucial mechanism for maintaining cellular integrity [30]. These findings underscore the importance of the ubiquitin-mediated proteolysis pathway in mitigating VC-related damage induced by heat stress.

The SUMO-activating enzyme SAE1, with the assistance of ATP, activates SUMO proteins [31]. Knocking down SAE1 in cancer cells has been shown to inhibit cell proliferation, which aligns with our findings that heat stress inhibits *SAE1* expression and consequently cell proliferation [32]. The *UBE3A* gene, encoding the ubiquitin E3-ligase protein UBE3A, is among the crucial E3 ligases in the ubiquitin–proteasome system. Deletion of *UBE3A* has been reported to enhance the growth capacity of mouse embryonic fibroblasts [33]. However, our study reveals that, under heat stress, *UBE3A* expression is upregulated, ultimately inhibiting cell proliferation. Upon the addition of the ubiquitin-mediated proteolysis pathway inhibitor ML323, we observed a significant downregulation of *UBE3A* expression in both the heat stress and VC treatment groups. Our findings demonstrate that exposure to high-temperature thermal stress significantly downregulates ubiquitin-mediated proteolysis in pig cells, whereas the addition of VC upregulates this pathway under conditions of heat stress. When ubiquitin was inhibited, the protective effects of VC against heat stress-induced apoptosis diminished, leading to reduced cell proliferation. These results highlight the critical importance of ubiquitin-mediated proteolysis in mediating VC’s ability to mitigate oxidative damage in pig cells subjected to heat stress.

In summary, this study demonstrates that 24 h of heat stress at 41 °C induces oxidative stress in porcine PTVC, leading to a reduced cell cycle progression, increased cell apoptosis, and consequently inhibiting cell proliferation. VC exerts its antioxidant effects through the ubiquitin-mediated proteolysis pathway, effectively mitigating the cellular damage caused by heat stress. These findings enhance our understanding of the mechanisms underlying the impact of ambient temperature on pig cells, offering novel insights into the protective role of VC and suggesting strategies to prevent heat stress-induced damage.

## Data Availability

The RNA sequencing datasets have been deposited in the NCBI short-read archive under BioProject no. PRJNA1155641.

## Supplementary Materials

The following supporting information can be downloaded at: https://www.mdpi.com/article/10.3390/antiox13111341/s1, Figure S1. EdU and ROS quantitative results; Figure S2. Network topology analysis was conducted to evaluate soft-thresholding powers ranging from 1 to 20 and to visualize the relationship between GS and MM alongside mRNA expression levels in green-yellow and red modules; Table S1. Primer sequences of gene selected for qRTP–CR; Table S2. Hub genes were identified in the green-yellow and red modules; Table S3. WGCNA Pathway Gene Enrichment Table.
