# Supplementary material for: Vitamin C Alleviates Heat-Stress-Induced Damages in Pig Thoracic Vertebral Chondrocytes via the Ubiquitin-Mediated Proteolysis Pathway
Source: Antioxidants (Basel). 2024 Nov 1;13(11):1341. doi: 10.3390/antiox13111341 (PMC11591123; doi:10.3390/antiox13111341)
Supplement: Supplementary file 1 [file antioxidants-13-01341-s001.zip › Vitamin C-supplementary figure.pdf]

Supplementary information

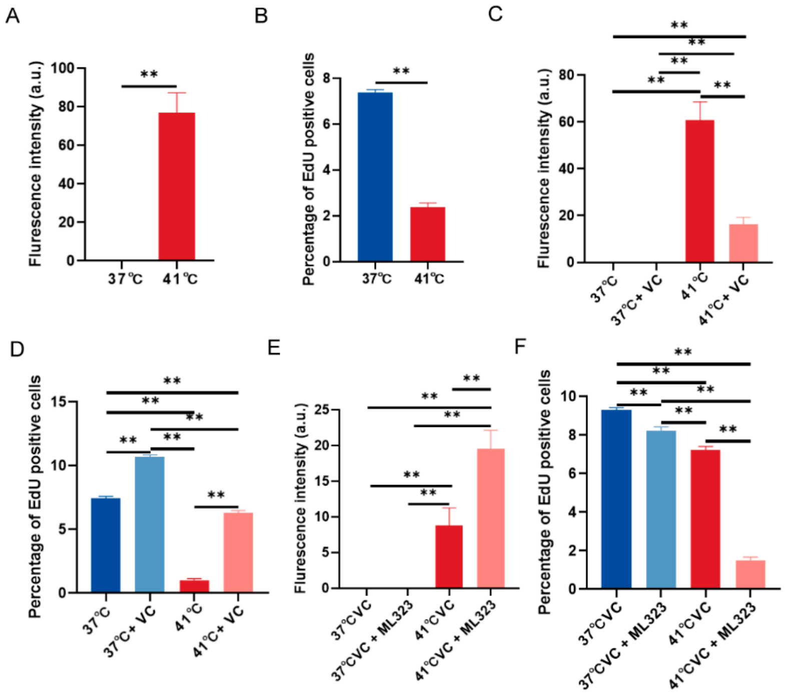

Figure S1. EdU and ROS quantitative results. (A) Quantitative fluorescence intensity of ROS in 37℃ and 41℃ groups. (B) The percentage of EdU-positive cells in 37℃ and 41℃ groups. (C) Quantitative fluorescence intensity of ROS in 37℃, 41℃, 37℃+ VC and 41℃ +VC groups. (D) The percentage of EdU-positive cells in 37℃, 41℃, 37℃+ VC and 41℃ +VC groups. (E) Quantitative fluorescence intensity of ROS in 37℃VC, 37℃VC + ML323, 41℃VC and 41℃VC + ML323 groups. (F) The percentage of EdU-positive cells in 37℃VC, 37℃VC + ML323, 41℃VC and 41℃VC + ML323 groups.

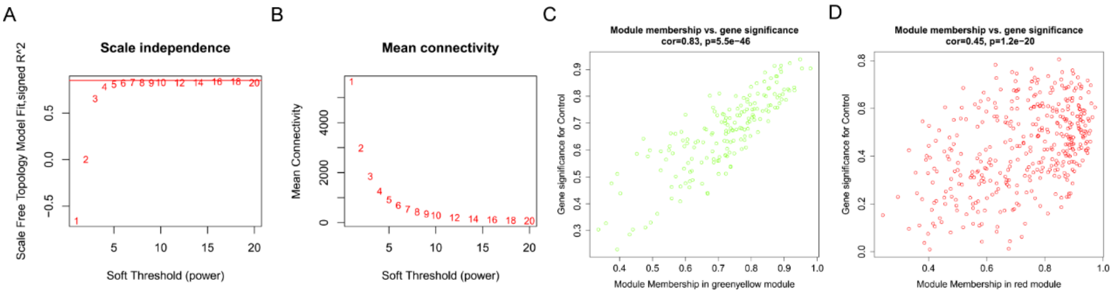

Figure S2. Network topology analysis was conducted to evaluate soft-thresholding powers ranging from 1 to 20 and to visualize the relationship between GS and MM alongside mRNA expression levels in greenyellow and red modules. (A) illustrates the effects of varying

soft-thresholding powers (x-axis) on the scale-free fit index (y-axis). (B) displays the impact of these soft-thresholding powers (x-axis) on mean connectivity (degree, y-axis). Scatterplots of GS for the differentiation stage versus MM in the (C) greenyellow and (D) red modules are also presented. In these plots, the correlation coefficient (cor) indicates the strength of the relationship between GS and MM, while the p-value signifies the statistical significance of this correlation.
